# Targeted Sequencing of Cancer-Related Genes in Colorectal Cancer Using Next-Generation Sequencing

**DOI:** 10.1371/journal.pone.0064271

**Published:** 2013-05-21

**Authors:** Sae-Won Han, Hwang-Phill Kim, Jong-Yeon Shin, Eun-Goo Jeong, Won-Chul Lee, Kyung-Hun Lee, Jae-Kyung Won, Tae-Yong Kim, Do-Youn Oh, Seock-Ah Im, Yung-Jue Bang, Seung-Yong Jeong, Kyu Joo Park, Jae-Gahb Park, Gyeong Hoon Kang, Jeong-Sun Seo, Jong-Il Kim, Tae-You Kim

**Affiliations:** 1 Department of Internal Medicine, Seoul National University Hospital, Seoul, Korea; 2 Cancer Research Institute, Seoul National University College of Medicine, Seoul, Korea; 3 Genomic Medicine Institute, Medical Research Center, Seoul National University, Seoul, Korea; 4 Psoma Therapeutics Inc., Seoul, Korea; 5 Department of Biomedical Sciences, Seoul National University Graduate School, Seoul, Korea; 6 Department of Pathology, Seoul National University Hospital, Seoul, Korea; 7 Department of Surgery, Seoul National University Hospital, Seoul, Korea; 8 Department of Biochemistry, Seoul National University College of Medicine, Seoul, Korea; 9 Macrogen Inc., Seoul, Korea; 10 Department of Molecular Medicine & Biopharmaceutical Sciences, Graduate School of Convergence Science and Technology, Seoul National University, Seoul, Korea; Tel Aviv University, Israel

## Abstract

Recent advance in sequencing technology has enabled comprehensive profiling of genetic alterations in cancer. We have established a targeted sequencing platform using next-generation sequencing (NGS) technology for clinical use, which can provide mutation and copy number variation data. NGS was performed with paired-end library enriched with exons of 183 cancer-related genes. Normal and tumor tissue pairs of 60 colorectal adenocarcinomas were used to test feasibility. Somatic mutation and copy number alteration were analyzed. A total of 526 somatic non-synonymous sequence variations were found in 113 genes. Among these, 278 single nucleotide variations were 232 different somatic point mutations. 216 SNV were 79 known single nucleotide polymorphisms in the dbSNP. 32 indels were 28 different indel mutations. Median number of mutated gene per tumor was 4 (range 0–23). Copy number gain (>X2 fold) was found in 65 genes in 40 patients, whereas copy number loss (<X0.5 fold) was found in 103 genes in 39 patients. The most frequently altered genes (mutation and/or copy number alteration) were *APC* in 35 patients (58%), *TP53* in 34 (57%), and *KRAS* in 24 (40%). Altered gene list revealed ErbB signaling pathway as the most commonly involved pathway (25 patients, 42%). Targeted sequencing platform using NGS technology is feasible for clinical use and provides comprehensive genetic alteration data.

## Introduction

Multiple genetic events accumulate during the progression of colorectal carcinogenesis [1]. There are a number of molecular subtypes of colorectal cancer including microsatellite instability and chromosomal instability [2], [3]. However, the subtypes have limited predictive or prognostic value and do not influence treatment decision in the metastatic setting. In contrast, *KRAS* mutation is the single most important and widely used molecular test in the metastatic colorectal cancer. Mutational status of *KRAS* guides treatment decision because presence of the mutation can predict lack of benefit from *EGFR*-targeted antibodies [4].

Large-scale sequencing analyses utilizing conventional sequencing methods has provided genetic landscape of colorectal cancer showing that there are a few gene “mountains” mutated in large proportion of tumors and many “hills” mutated infrequently [5], [6]. In addition, genome-wide copy number analysis revealed that colorectal cancer has fewer copy number alterations compared with breast cancer [7]. Recent advance in the sequencing technology using next-generation sequencing (NGS) has facilitated analysis of entire genome in individual cancers and identification of novel genetic alterations [8], [9]. In studies of colorectal cancer, novel recurrent genetic fusions have been identified [10], [11]. However, major shortcoming of whole genome or exome sequencing approach is identification of many functionally unclear, uncommon, possible “passenger” mutations with unknown clinical significance. Moreover, relatively low coverage of the large-scale approach has limitation in sensitivity and specificity, which is an important issue for application in clinical setting. Genetic alterations identified by low coverage analysis need confirmation to be used in clinical decision making.

Targeted sequencing could be a better alternative for clinical application of NGS technology. Advantage of targeted approach is increase in coverage depth compared to whole exome approach by reducing the number of genes analyzed with similar number of base pairs sequenced. This enables generation of reliable data with sufficient sequencing depth in the targeted genes of interest.

We have established a targeted sequencing platform using NGS technology, which includes 183 genes and provides mutation and copy number variation data. The purpose of this study was to test the feasibility of the targeted sequencing platform for future clinical application using colorectal tumor tissues.

## Materials and Methods

### Ethics statement

The study protocol was reviewed and approved by the Institutional Review Board of Seoul National University Hospital (SNUH). All patients gave written informed consent for tissue banking and genetic testing prior to surgery. This study was carried out in accordance with the recommendations of the Declaration of Helsinki for biomedical research involving human subjects.

### Study overview

A total of 183 genes (Table S1) were selected with following criteria: known to predict response, therapeutically targetable, involved in major signaling pathways, and high mutation frequency in the Catalogue of Somatic Mutations in Cancer database (COSMIC). Fresh frozen primary tumor and adjacent normal tissue specimens were acquired from SNUH Tumor Bank. The specimens were deidentified and clinico-pathologic information was provided by the Tumor Bank. DNA extracted from the tissue was sent to Genome Medicine Institute, Seoul National University. Sequencing results were reported within 3 weeks (Figure S1).

### Target enrichment of genomic DNA and sequencing

Genomic DNA was extracted from the paired specimens using the QIAamp DNA Mini kit (Qiagen, Hilden, Germany). Three micrograms of DNA was sheared using a Covaris S2 (Covaris, Inc., Massachusetts, USA) to ∼250 nt at a 20% duty cycle, level 5 intensity and 200 cycles per burst for 180 s. Bar-coded fragment sequencing libraries were made using an Paired-end DNA sample preparation kit (Illumina, California, USA) and Illumina multiplexing adaptor (Illumina) according to the manufacturer's instructions. After ligation with the Illumina adaptor, the libraries were prepared using AMPure bead (Beckman Coulter, Inc., California, USA) rather than gel purification. Library quality was assessed using an Agilent 2100 Analyser and DNA 1000 chips (Agilent Technology, California, USA). To design the RNA baits for capture, we utilized the COSMIC (http://www.sanger.ac.uk/genetics/CGP/cosmic) and the Agilent Technologies eArray site (https://earray.chem.agilent.com/earray/). The targeted regions included all exons of 183 genes involved in various cancers and total length captured was ∼1 M. The baits were 120 bp long, and the average bait coverage of each base in the target region was 2X. We avoided standard repeat masked regions but allowed each bait to overlap with a repetitive region up to 20 bp. We also identified sequences within repetitive regions that were sufficiently unique to serve as reasonable baits. An equimolar eight-plex pool was produced for enrichment using a SureSelect Target Enrichment System Kit (Agilent Technology) and a modified protocol. Five hundred nanograms of pooled DNA with 5 µl (100 ng) of custom baits were used for enrichment, with blocking oligonucleotides specific for paired-end sequencing libraries and 24-h hybridization. Biotinylated RNA library hybrids were recovered with streptavidin beads. The captured libraries were amplified and sequenced on the Illumina Genome Analyser IIx by 2×69 cycles. We aligned the resulting short-sequence reads to the reference genome (NCBI human genome assembly build 37) using the Genomic Short-read Nucleotide Alignment Program (GSNAP) alignment program [12], with allowance for 5% mismatches after accounting for PCR duplicates and reads that did not align to captured regions of the reference genome. The sequencing data are uploaded to the EBI European Nucleotide Archive (http://www.ebi.ac.uk/ena/home) under accession number ERP002442.

### Single nucleotide variant (SNV) and indel detection

We called genomic variants of each sample (SNVs and short indels) using modified criteria from our previous publications in whole-genome sequencing [13], [14]. Briefly, SNVs and indels were defined based on satisfaction of the following three conditions: (1) the number of uniquely mapped reads at the position should be two or more; (2) the average base quality (*phred* Q score) for the position should be 20; and (3) the read-allele frequency at the position should be 20%. For detection of the somatic mutations (SNVs and indels) in the cancer tissues, we used the following conditions: (1) nonsynonymous SNVs or indels in the cancer tissues; (2) the SNV allele count should be zero in targeted sequence of normal tissue; (3) the wild-type allele count should be 10 or more in targeted sequence of normal tissue; and (4) the candidate positions should not be polymorphisms according to the dbSNP132. The functional consequences of the novel missense variants were predicted using Sorting Intolerant from Tolerant (SIFT) [15]. Mutation in *KRAS* was confirmed using Sanger sequencing. Following primers were used: codon12 and 13, forward 5′-CGTCTGCAGTCAACTGGAAT-3′ and reverse 5′-GAGAGTGAACATCATGGACC-3′; codon 61, forward 5′-CAGACTGTGTTCTCCCTTCTCA-3′ and reverse 5′-CTCATGTACTGGTCCCTCATTG-3′; and codon 146, forward 5′-TGGACAGGTTTTGAAAGATATTTG-3′ and reverse 5′-ATTAAGAAGCAATGCCCTCTCAAG-3′. All sequencing reactions were done in both forward and reverse directions, and all mutations were confirmed at least twice from independent PCR isolates.

### Copy number alteration

We estimated the coverage of genes by using sequencing reads mapped to the targeted regions. To detect copy number alteration for a given pair of cancer and normal tissues, coverage fold ratios (tumor/normal) for 183 target genes were calculated. After the normalization step considering total read bases obtained from each tissue, coverage fold ratio was adjusted by estimated tumor cell purity described below. We defined that a gene shows copy number gain when its coverage fold ratio ≥2.0 and loss when ≤0.5.

To estimate tumor purity, we used read counts of somatic SNVs identified. Given somatic SNVs for each cancer tissue, we assumed that the cancer consists of a major clone and the SNVs are derived from the clone. Moreover, we regarded the SNVs as heterozygotes whose allele frequency is 0.5. With these assumptions, tumor purity was estimated as follows. The expected numbers of wild-type reads originated from cancer clone and normal cells were calculated. Then, the proportion of wild-type plus SNP read counts for cancer among the total number of read counts was considered as the corresponding tumor purity. In 3 samples that had no tumor specific SNV, the median value of 57 samples was used in the analysis of copy number variations.

Copy number alteration was confirmed using quantitative real-time PCR with the iCycler IQ detection system (Bio-Rad Laboratories, Hercules, CA) using SYBR green I (Molecular Probe, Eugene, OR) in triplicate reactions. The primers used in the PCR reaction were as following: *ERBB2*, forward 5′-TGCTGGAGGACGATGACATG-3′ and reverse 5′-CTGGACAGAAGAAGCCCTGC-3′; *SRC*, forward 5′- CGGTTACTGCTCAATGCAGA-3′ and reverse 5′-CAAGAGCGCTCGTACCTTTC-3′; *TP53*, forward 5′-CCCTTCCCAGAAAACCTACC-3′ and reverse 5′- ACTGACCGTGCAAGTCACAG-3′; *PTEN*, forward 5′- TGGCACTGTTGTTTCACAAG-3′ and reverse 5′- TGTTCCAATACATGGAAGGATG-3′; *BRCA1*, forward 5′- GTTTGCCAGAAAACACCACA-3′ and reverse 5′-TTTATGCAGCAGATGCAAGG -3′; *BRCA2*, forward 5′-TGCCTGGCCTGATACAATTA-3′ and reverse 5′- TTGCTGCTTCCTTTTCTTCC-3′; and *ACTB*, forward 5′- AGAGCTACGAGCTGCCTGAC and reverse 5′- GGATGCCACAGGACTCCA-3′. Fluorescence *in situ* hybridization (FISH) analysis of *ERBB2*/CEP17 was performed using the PathVysion kit (Vysis, Downers Grove, IL) according to the manufacturer's instructions.

### Microsatellite status

The microsatellite status of each tumor was determined by examining 5 microsatellite markers (D2S123, D5S346, D17S250, BAT25, and BAT26) as previously described [16]. Either forward or reverse primer for each marker was labeled with fluorescence, and PCR products were electrophoresed and analyzed. We classified MSI status as follows: MSI-high, instability at two or more microsatellite markers, MSI-low, instability at one marker, and MSS, no instability marker.

### Pathway analysis

Pathway analysis was performed for genes having mutation or copy number alteration in each tumor using Database for Annotation, Visualization and Integrated Discovery (DAVID) Bioinformatics Resources version 6.7 utilizing the pathway databases BBID, Biocarta, and Kyoto Encyclopedia of Genes and Genomes (KEGG) [17]. Functional annotation chart in DAVID was created using the human genome as background, and thresholds for count as 2 and EASE score as 0.1.

## Results

### Patient characteristics and sequencing profile

Patient characteristics are as described in Table 1. Paired specimens of primary colorectal tumor and adjacent normal mucosa removed during operation were used for analysis.

**Table 1:** Baseline characteristics.

Characteristics		Number (%)
Sex	Male	40 (67)
	Female	20 (33)
Age	Median (range)	63.5 (35–86)
Site	Ascending colon	18 (30)
	Descending colon	5 (8)
	Sigmoid colon	14 (23)
	Rectum	23 (38)
Stage	1	7 (12)
	2	27 (45)
	3	19 (32)
	4	7 (12)
Microsatellite instability	MSS	48 (80)
	MSI-L	6 (10)
	MSI-H	6 (10)

Average coverage of the 120 samples analyzed was175X (range 99X–435X) (Table S2). It was 181X (100X–435X) for tumor and 170X (99X–312X) for normal mucosa. Tumor cell percentage in the tumor specimen could be estimated using tumor-specific SNVs in 57 samples. The median percentage was 71% (range 42–100).

### Mutations

**Figure 1:**
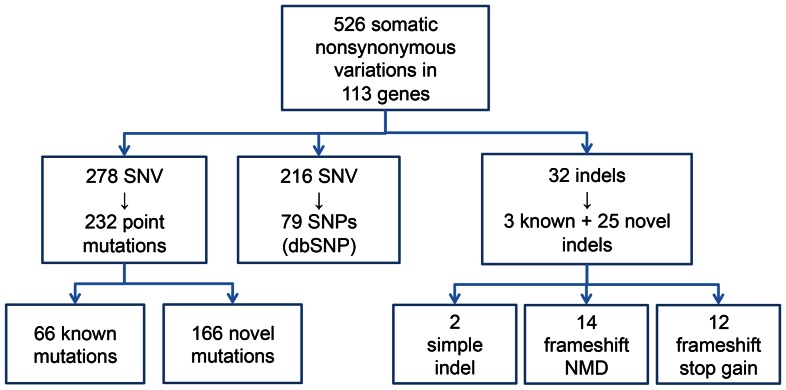
Summary of mutations. SNV, single nucleotide variation; SNP, single nucleotide polymorphism; NMD, non-sense mediated decay.

A total of 532 somatic nonsynonymous variations in 113 genes were found (Figure 1). 494 single nucleotide variations were observed in 106 genes from 60 patients and 38 indel mutations (11 insertions and 27 deletions) in 19 genes from 21 patients (Table S3).

Among the 494 SNVs, 278 variations were 232 different point mutations (67 previously reported mutations and 166 novel mutations) and 216 were previously reported 79 different polymorphisms in the dbSNP.

Point mutations comprised of 43 nonsense mutations and 189 missense mutations. We found 2 recurrent novel mutations, *JAK1* c.1595C>T (p.R532H) in 2 patients and *EWSR1* c.1769A>C (p.Q590P) in 2. Point mutations were most frequently observed in *APC* (32 mutations in 29 patients), followed by *TP53* (27 in 27), *KRAS* (24 in 24), *TTN* (36 in 21), and *FBXW7* (15 in 14) (Table 2).

**Table 2:** Most commonly altered genes.

	Point mutation*	Indel	No. of patients with mutation (%)	CNV**	No. of patients with mutation or CNV (%)
***APC***	32 (29)	7 insertion, 2 deletion	35 (58)	3 loss	35 (58)
***TP53***	27 (27)	1 deletion	27 (45)	13 loss	34 (57)
***KRAS***	24 (24)	0	24 (40)	0	24 (40)
***TTN***	36 (21)	0	21 (35)	0	21 (35)
***FBXW7***	15 (14)	1 deletion	15 (25)	2 loss	17 (28)
***SMAD4***	3 (3)	1 insertion, 1 deletion	5 (8)	12 loss	17 (28)
***MAFB***	1 (1)	0	1 (2)	15 gain	16 (27)
***GNAS***	4 (4)	0	4 (7)	13 gain	16 (27)
***SRC***	0 (0)	0	0 (0)	15 gain	15 (25)

* Number of mutations (number of patients).

** Gain > x2.0, loss < x0.5.

The 32 indel mutations were 3 known mutations and 25 novel mutations, which were 2 simple deletions of an amino acid, 26 frameshift mutations. Among the frameshift mutations, 14 are predicted to result in nonsense mediated decay. 3 novel indel mutations were recurrently observed: *ACVR2A* c.1303delA (p.K435fs) in 3, *TGFBR2* c.449_450delAA (p.E150fs) in 2, and *BLM* c.1536delA (p.G512fs) in 2.

Median numbers of mutation and mutated gene per tumor were 4.5 (range 0–32) and 4.0 (0–23), respectively. Most commonly mutated genes were *APC* (35 patients), *TP53* (27), and *KRAS* (24). We performed Sanger sequencing for validation of *KRAS* mutation and all mutations identified by NGS was confirmed.

### Copy number alterations

**Figure 2:**
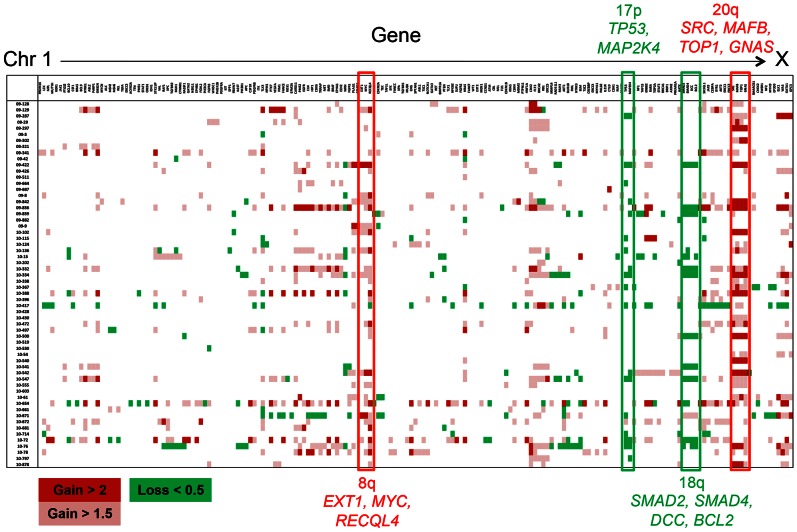
Copy number variation table. Samples are aligned according to sample identification number in the Y-axis and genes are aligned according to chromosomal location in the X-axis.

**Figure 3:**
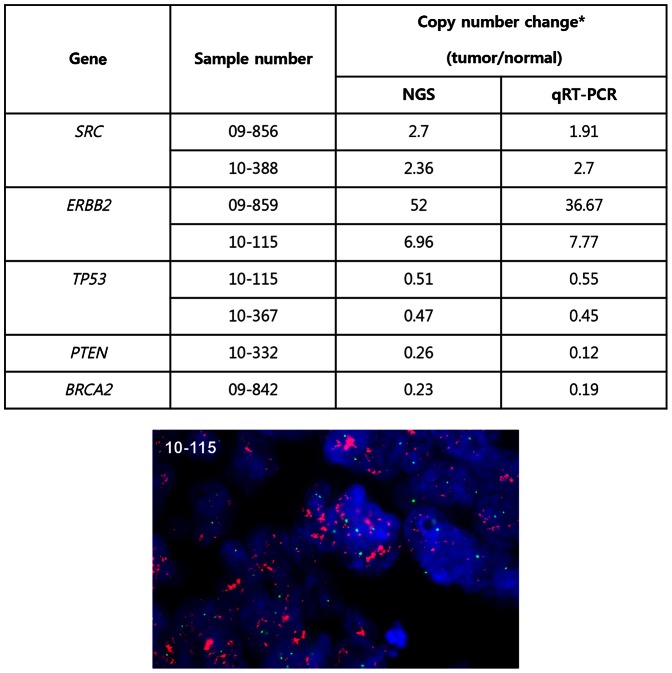
Validation of copy number variation. * Values are coverage fold ratios not adjusted for tumor purity. Representative image of FISH analysis of *ERBB2* (red) and CEP17 (green) showing amplification of *ERBB2*.

Copy number change was observed in 132 genes and 51 tumors (85%) (Figure 2 and Table S4). Copy number gain (>X2 fold) was found in 65 genes from 40 tumors and copy number loss (<X0.5 fold) in 103 genes from 39 tumors. Among the tumors showing copy number change, median number of genes involved was 6 (range 1–44). Genes most frequently showing copy number gain were *SRC* (15 patients), *MAFB* (15), *TOP1* (14), *RECQL4* (13), and *GNAS* (13). Except for *RECQL4* located at chromosome 8q24, the other 4 genes were located at 20q 11–13. Genes showing frequent loss were *TP53* (13 patients), *SMAD2* (12), *SMAD4* (12), *MAP2K4* (11), *BCL2* (11), *WRN* (10), and *DCC* (10). *TP53* and *MAP2K4* were located at 17p12–13, whereas *SMAD2*, *SMAD4*, *BCL2*, and *DCC* were located at 18q21, and *WRN* located at 8p12. Quantitative RT-PCR was used for validation of copy number alteration of *SRC*, *ERBB2*, *TP53*, *PTEN* and *BRCA2* in representative samples and showed comparable values. *ERBB2* was further validated using FISH (Figure 3).

### Combined genetic alterations

Combining mutation and copy number data, *APC* was the gene most frequent genetic alteration (35 patients). Three patients had copy number loss of *APC*, but the 3 tumors also had indel mutations. In case of *TP53*, 6 patients concomitantly had point mutation and copy number loss and 7 patients had copy number loss as the only genetic mechanism of *TP53* inactivation. There was no copy number change in *KRAS*. However, copy number gain of *HRAS* was found in 6 patients.

MSI-H tumors tended to have higher number of genes with mutation compared with MSS/MSI-L (mean 10.8 *vs*. 3.9, respectively; *p* = 0.11 by t-test) and lower number of genes having copy number alteration (mean 3.2 *vs.* 8.6, respectively; *p* = 0.22 by t-test). MSI-H tumors had higher frequencies of genetic alterations in *ACVR2A* (1.9% in MSS/MSI-L *vs.* 50.0% in MSI-H, *p* = 0.002), *MLH1* (3.7% *vs.* 33.3%, *p* = 0.046), *MSH6* (1.9% *vs.* 33.3%, *p* = 0.024), *SPTAN1* (0% *vs.* 33.3%, *p* = 0.008), and *TGFBR2* (1.9% *vs.* 33.3%, *p* = 0.024). In contrast, genetic alterations of *TP53* (63.0% *vs.* 0%, *p* = 0.005) were more frequently observed in MSS/MSI-L tumors.

We could also identify genetic alterations that could have potential therapeutic implications (Table 3). *ERBB2* copy number gain was identified in 4 patients (range X2.0–X71.5) and *ERBB2* point mutation in 5 patients (1 patient had copy number gain and point mutation). Genetic alterations in *BRCA1* and *BRCA2* were observed in 4 patients: *BRCA1* loss (X0.49), *BRCA1* point mutation, *BRCA2* loss (X0), and *BRCA2* deletion mutation in 1 patient each. *EGFR* copy number gain was found in 4 patients (range X2.0–8.2). *PIK3CA* point mutation was observed in 4 patients and *PTEN* loss and point mutation in 4 patients (range X0–X0.49) and 1 patient, respectively.

**Table 3:** Genetic alterations with potential therapeutic implication.

Candidate treatment	Genetic alteration (No. of patients)
ERBB2 directed treatment	*ERBB2* copy number gain (4)
	*ERBB2* mutation (5)
EGFR directed treatment	*EGFR* copy number gain (4)
PI3K inhibitor	*PIK3CA* mutation (4)
	*PTEN* copy number loss (4)
	*PTEN* mutation (1)
PARP inhibitor	*BRCA1* copy number loss (1)
	*BRCA1* mutation (1)
	*BRCA2* copy number loss (1)
	*BRCA2* mutation (1)

### Pathway analysis

A total of 33 patients (55%) had possible gain-of-function alteration in the *RAS*/*RAF* pathway: 20 patients with *KRAS* mutation only, 3 with *KRAS* mutation and gain of *HRAS*, 1 with *KRAS* mutation and *BRAF* mutation, 3 with *NRAS* mutation, 2 with *HRAS* gain, 2 with *BRAF* mutation, 1 with *BRAF* mutation and *HRAS* gain, and 1 with *RAF1* gain.

Analyzing list of altered genes (mutation and copy number change) using DAVID functional annotation tools, related pathway was identified in 53 patients (Table S5). Median number of pathway per patient was 19 (range 3–70). Excluding disease-related pathways (e.g, colorectal cancer), ErbB signaling pathway (KEGG hsa04012) was the most frequently involved pathway (25 patients, 42%).

## Discussion

In the coming era of personalized medicine for cancer treatment, it is essential to have exact genetic information of the individual cancer. Number of genetic information already guides treatment decisions in daily practice. Examples in treatment of solid tumors include *ERBB2* (HER2) gene amplification in breast and gastric cancer, *EGFR* mutation in non-small cell lung cancer, and *KRAS* mutation in colorectal cancer [18], [19], [20], [21]. Personalized cancer treatment is pursued from early stage of drug development when there is a known target [22]. However, many of the targeted agents under development do not have a predictive genetic biomarker. Thorough information of genetic status of patients enrolled into early phase clinical trials and analysis of association with response may accelerate target patient identification.

We have established a targeted sequencing platform using NGS technology to provide comprehensive genetic information for individual patients. In the present study, we show that NGS-based targeted sequencing platform is feasible for clinical use. Even though we did not confirm every genetic alteration identified, validation experiment of *KRAS* mutation and representative copy number alterations (Figure 3) shows that the results of the platform is reliable. In addition, the profile of commonly mutated genes and pattern of gene copy number alteration (gains of 8q and 20q and losses of 8p, 17p and 18q) are consistent with prior knowledge of genetic alterations in colorectal cancer [23].

Major advantage of the NGS-based targeted sequencing platform is that data regarding multiple genes and multiple genetic alterations (point mutation, indel mutation, and copy number alteration) is generated with a single experiment. The sequencing platform providing mutation and copy number alteration data requires less amount of DNA or tissue and cost compared with testing individual genetic alteration by sequencing or FISH which are commonly used in current daily practice. Moreover, superior sensitivity over Sanger sequencing can be obtained by increasing coverage depth, especially in cases with low tumor purity.

In addition to *KRAS* mutation, mutation of other genes in the pathway (*BRAF*, *NRAS*, and *PIK3CA*) also has negative effect on response to cetuximab [24]. It is likely that examining multiple genes in the pathway could also improve response prediction in other targeted treatments. Targeted sequencing platform is an ideal option to evaluate multiple genes at the same time. In addition to *KRAS* mutation, genetic alterations in *NRAS*, *HRAS*, *BRAF*, and *RAF1* were found in colorectal cancer samples analyzed in this study.

Finding uncommon genetic alteration may provide new treatment option for individual patient. For example, patients with tumors having *ERBB2* copy number gain may benefit from *ERBB2*-targeted agents and tumors having mutation or loss of *BRCA1* or *BRCA2* from PARP inhibitors. Moreover, genetic alterations or involved pathway may guide selection of early phase clinical trial for patients to be enrolled. Patients with alteration of *RAS/RAF* pathway might have more chance of benefit by participating in a clinical trial of inhibitors targeting the pathway.

In order to detect multiple mutations with enhanced sensitivity, mass spectrometry based mutation detection platform is currently in use [25]. However, only limited number of pre-specified mutations can be detected and copy number alteration cannot be detected with the platform. Therefore, targeted sequencing platform using NGS is superior in terms of the genetic information produced and flexibility of constituting gene sets for analysis. Recent studies have also shown usefulness of targeted sequencing approach or NGS in the clinical setting [9], [26], [27].

Major limitation of the present study is that the platform is unable to detect fusion genes and only fresh frozen tissue was used for analysis. Detection of fusion genes could be enabled by addition of low coverage RNA sequencing. We have modified experimental procedure to utilize formalin-fixed paraffin-embedded (FFPE) tissue and have obtained comparable sensitivity. We are currently studying the usefulness of the targeted sequencing platform in a prospective manner in clinical practice. The study utilizes either fresh or FFPE tissue and we have modified gene list to better represent druggable pathways and increased coverage depth (>X500).

In conclusion, targeted sequencing platform using NGS technology was able to provide comprehensive genetic alteration data in colorectal tumor samples and can be used in clinical setting.

## Supporting Information

Figure S1 **Study outline.**
(TIF)

Table S1 Gene list.
(XLSX)

Table S2 Coverage information.
(XLSX)

Table S3 Genetic alterations per patients.
(XLSX)

Table S4 Copy number alteration.
(XLSX)

Table S5 DAVID pathways.
(XLSX)
