# Supplementary figures and images for: Targeted Sequencing of Cancer-Related Genes in Colorectal Cancer Using Next-Generation Sequencing
Source: PLoS One. 2013 May 21;8(5):e64271. doi: 10.1371/journal.pone.0064271 (PMC3660257; doi:10.1371/journal.pone.0064271)

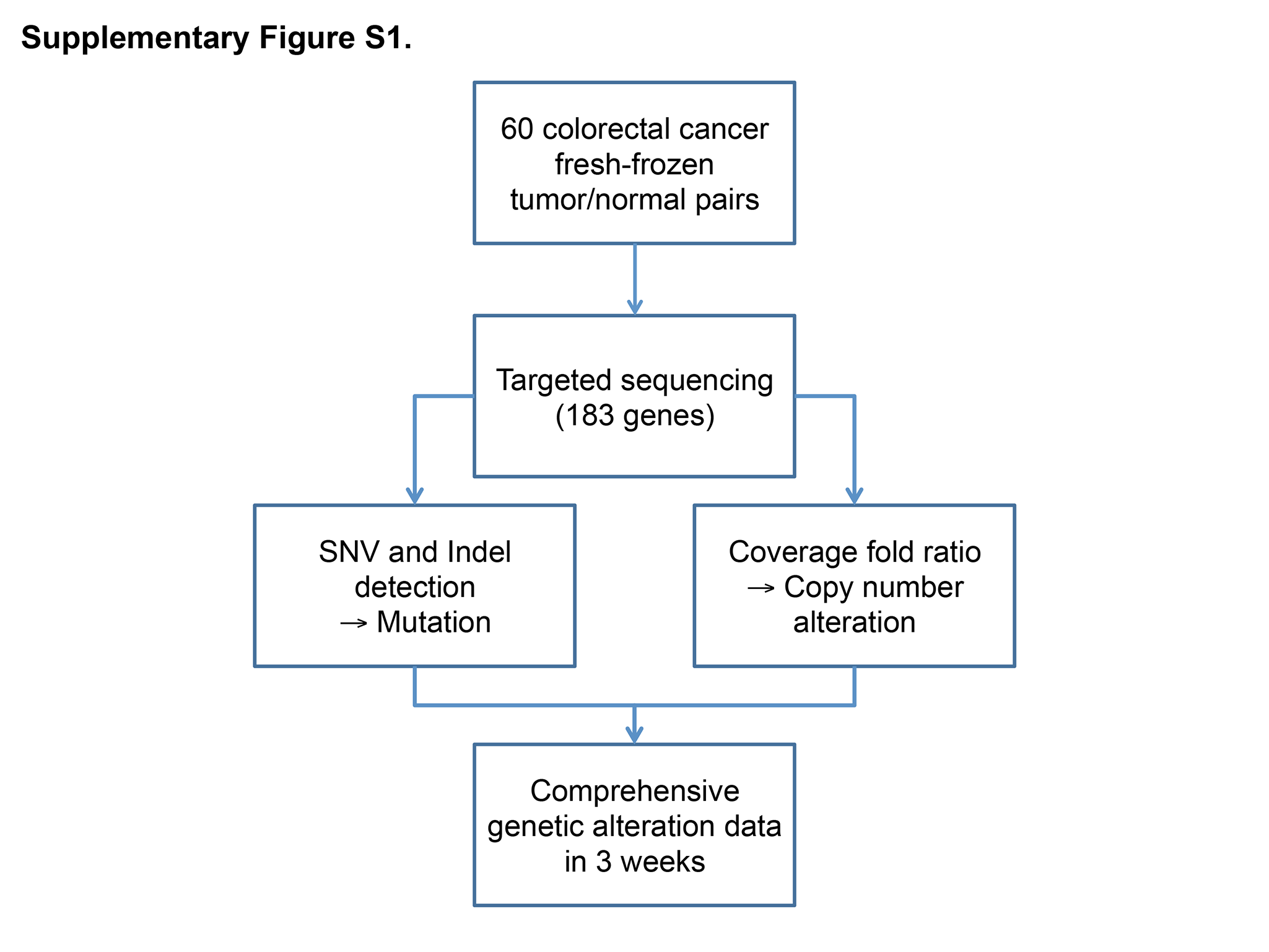

Supplement: Figure S1 — Study outline. (TIF) [file pone.0064271.s001.tif]
